# Born Too Soon: The global epidemiology of 15 million preterm births

**DOI:** 10.1186/1742-4755-10-S1-S2

**Published:** 2013-11-15

**Authors:** Hannah Blencowe, Simon Cousens, Doris Chou, Mikkel Oestergaard, Lale Say, Ann-Beth Moller, Mary Kinney, Joy Lawn

**Affiliations:** 1 MARCH, London School of Hygiene and Tropical Medicine, London, UK; 2 World Health Organization, Geneva, Switzerland; 3 Saving Newborn Lives, Save the Children, Cape Town, South Africa

**Keywords:** Preterm birth, epidemiology, neonatal mortality

## Abstract

This second paper in the Born Too Soon supplement presents a review of the epidemiology of preterm birth, and its burden globally, including priorities for action to improve the data. Worldwide an estimated 11.1% of all livebirths in 2010 were born preterm (14.9 million babies born before 37 weeks of gestation), with preterm birth rates increasing in most countries with reliable trend data. Direct complications of preterm birth account for one million deaths each year, and preterm birth is a risk factor in over 50% of all neonatal deaths. In addition, preterm birth can result in a range of long-term complications in survivors, with the frequency and severity of adverse outcomes rising with decreasing gestational age and decreasing quality of care. The economic costs of preterm birth are large in terms of immediate neonatal intensive care, ongoing long-term complex health needs, as well as lost economic productivity. Preterm birth is a syndrome with a variety of causes and underlying factors usually divided into spontaneous and provider-initiated preterm births. Consistent recording of all pregnancy outcomes, including stillbirths, and standard application of preterm definitions is important in all settings to advance both the understanding and the monitoring of trends. Context specific innovative solutions to prevent preterm birth and hence reduce preterm birth rates all around the world are urgently needed. Strengthened data systems are required to adequately track trends in preterm birth rates and program effectiveness. These efforts must be coupled with action now to implement improved antenatal, obstetric and newborn care to increase survival and reduce disability amongst those born too soon.

**Declaration:**

This article is part of a supplement jointly funded by Save the Children's Saving Newborn Lives programme through a grant from The Bill & Melinda Gates Foundation and March of Dimes Foundation and published in collaboration with the Partnership for Maternal, Newborn and Child Health and the World Health Organization (WHO). The original article was published in PDF format in the WHO Report "Born Too Soon: the global action report on preterm birth" (ISBN 978 92 4 150343 30), which involved collaboration from more than 50 organizations. The article has been reformatted for journal publication and has undergone peer review according to *Reproductive Health*'s standard process for supplements and may feature some variations in content when compared to the original report. This co-publication makes the article available to the community in a full-text format.

## Why focus on preterm birth?

**Figure 1:**
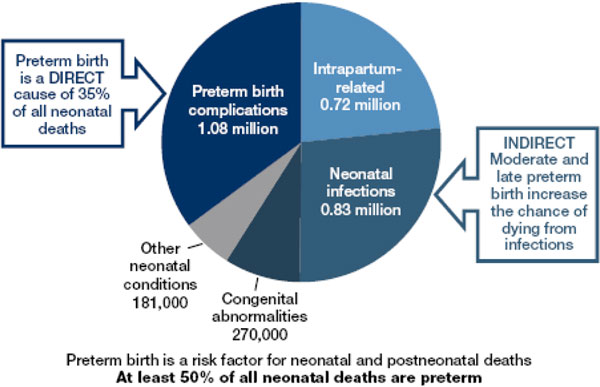
**Estimated distribution of causes of 3.1 million neonatal deaths in 193 countries in 2010**. Source: Updated from Lawn et al., 2005, using data from 2010 published in Liu L, et al., 2012.

Preterm birth is a major cause of death and a significant cause of long-term loss of human potential amongst survivors all around the world. Complications of preterm birth are the single largest direct cause of neonatal deaths, responsible for 35% of the world's 3.1 million deaths a year, and the second most common cause of under-5 deaths after pneumonia (Figure 1). In almost all high- and middle-income countries of the world, preterm birth is the leading cause of child death [1]. Being born preterm also increases a baby's risk of dying due to other causes, especially from neonatal infections [2] with preterm birth estimated to be a risk factor in at least 50% of all neonatal deaths [3].

Addressing preterm birth is essential for accelerating progress towards Millennium Development Goal 4 [4,5]. In addition to its significant contribution to mortality, the effect of preterm birth amongst some survivors may continue throughout life, impairing neuro-developmental functioning through increasing the risk of cerebral palsy, learning impairment and visual disorders and affecting long-term physical health with a higher risk of disease [6]. These effects exert a heavy burden on families, society and the health system (Table 1) [7,8]. Hence, preterm birth is one the largest single conditions in the Global Burden of Disease analysis given the high mortality and the considerable risk of lifelong impairment [9].

**Table 1:** Long-term impact of preterm birth on survivors

Long-term outcomes		Examples:	Frequency in survivors:
**Specific physical effects**	Visual impairment	• Blindness or high myopia after retinopathy of prematurity • Increased hypermetropia and myopia	Around 25% of all extremely preterm affected[80] Also risk in moderately preterm babies especially if poorly monitored oxygen therapy
	Hearing impairment		Up to 5 to 10% of extremely preterm[81]
	Chronic lung disease of prematurity	• From reduced exercise tolerance to requirement for home oxygen •Increased hospital admissions in childhood for LRTI[82]	Up to 40% of extremely preterm[83]
	Long-term cardiovascular ill-health and non-communicable disease	• Increased blood pressure • Reduced lung function • Increased rates of asthma • Growth failure in infancy, accelerated weight gain in adolescence	Full extent of burden still to be quantified
**Neuro-developmental/ behavioral effects**[84]	Mild Disorders of executive functioning	• Specific learning impairments, dyslexia, reduced academic achievement	
	Moderate to severe Global developmental delay	• Moderate/severe cognitive impairment • Motor impairment • Cerebral palsy	Affected by gestational age and quality of care dependent[85]
	Psychiatric/ behavioral sequelae	• Attention deficit hyperactivity disorder • Increased anxiety and depression	
**Family, economic and societal effects**	Impact on family Impact on health service Intergenerational	• Psychosocial, emotional and economic • Cost of care[7] - acute, and ongoing • Risk of preterm birth in offspring	Common varying with medical risk factors, disability, socioeconomic status[86]

Data on preterm birth rates are not routinely collected in many countries and, where available, are frequently not reported using a standard international definition. Time series using consistent definitions are lacking for all but a few countries, making comparison within and between countries challenging. In high-income countries with reliable data, despite several decades of efforts, preterm birth rates appear to have increased from 1990 to 2010 [10-12], although the United States reports a slight decrease in the rates of late preterm birth (34 to <37 completed weeks) since 2007 [13].

Recent estimates of preterm birth rates (all live births before 37 completed weeks) for 184 countries in 2010 and a time series for 65 countries with sufficient data suggest that 14.9 million (uncertainty range: 12.3-18.1 million) babies were born preterm in 2010 [14]. This paper reviews the epidemiology of preterm birth, and makes recommendations for efforts to improve the data and use the data for action to address preterm birth.

## Understanding the data

### Preterm birth -- what is it?

#### Defining preterm birth

**Figure 2:**
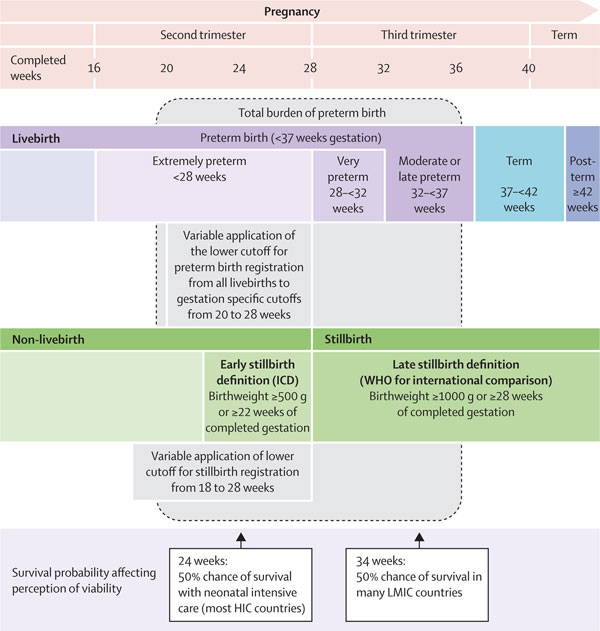
**Overview of definitions for preterm birth and related pregnancy outcomes**. Source: Reproduced with permission from Blencowe et al. (2012) National, regional and worldwide estimates of preterm birth rates in the year 2010 with time trends since 1990 for selected countries: a systematic analysis and implications. Lancet **379**(9832): 2162-2172.

Preterm birth is defined by WHO as all births before 37 completed weeks of gestation or fewer than 259 days since the first day of a woman's last menstrual period [15]. Preterm birth can be further sub-divided based on gestational age: extremely preterm (<28 weeks), very preterm (28 - <32 weeks) and moderate preterm (32 - <37 completed weeks of gestation) (Figure 2). Moderate preterm birth may be further split to focus on late pre-term birth (34 - <37 completed weeks). The 37 week cut off is somewhat arbitrary, and it is now recognized that whilst the risks associated with preterm birth are greater the lower the gestational age, even babies born at 37 or 38 weeks have higher risks than those born at 40 weeks gestation [16].

The international definition for stillbirth rate clearly states to use stillbirths > 1,000 g or 28 weeks gestation, improving the ability to compare rates across countries and times [17,18]. For preterm birth, International Classification of Disease (ICD) encourages the inclusion of all live births. This definition has no lower boundary, which complicates the comparison of reported rates both between countries and within countries over time since perceptions of viability of extremely preterm babies change with increasingly sophisticated neonatal intensive care, and some countries only include live births after a specific cut-off, for example, 22 weeks. In addition, other reports use non-standard cut-offs for upper gestational age (e.g., including babies born at up to 38 completed weeks of gestation).

In many high-and middle-income countries, the official definitions of live birth or stillbirth have changed over time. Even without an explicit lower gestational age cut-off in national definitions, the medical care given and whether or not birth and death registration occurs may depend on these perceptions of viability [19,20]. Hence, even if no "official" lower gestational age cut-off is specified for recording a live birth, misclassification of a livebirth to stillbirth is more common if the medical team perceives the baby to be extremely preterm and thus less likely to survive [20]. Eighty percent of all stillbirths in high-income countries are born preterm, accounting for 5% of all preterm births. Counting only live births underestimates the true burden of preterm birth [21,22].

In addition to the definition and perceived viability issue, some reports include only singleton live births, complicating comparison even further. From a public health perspective and for the purposes of policy and planning, the total number of preterm births is the measure of interest.

### Preterm birth - why does it occur?

Preterm birth is a syndrome with a variety of causes which can be classified into two broad subtypes: (1) *spontaneous *preterm birth (spontaneous onset of labour or following prelabour premature rupture of membranes (pPROM)) and (2) *provider-initiated *preterm birth (defined as induction of labor or elective caesarean birth before 37 completed weeks of gestation for maternal or fetal indications (both "urgent" or "discretionary"), or other non-medical reasons) (Table 2) [23].

**Table 2:** Types of Preterm Birth and Risk Factors

Type:	Risk Factors:	Examples:	Interventions:*
**Spontaneous preterm birth:**	Age at pregnancy and pregnancy spacing	Adolescent pregnancy, advanced maternal age, or short inter-pregnancy interval	Preconception care, including encouraging family planning beginning in adolescence and continuing between pregnancies
	Multiple pregnancy	Increased rates of twin and higher order pregnancies with assisted reproduction	Introduction and monitoring of policies for best practice in assisted reproduction
	Infection	Urinary tract infections, asymptomatic bactiuria, malaria, HIV, syphilis, chorioamnionitis, bacterial vaginosis	Sexual health programs aimed at prevention and treatment of infections prior to pregnancy. Specific interventions to prevent infections and mechanisms for early detection and treatment of infections occurring during pregnancy.
	Underlying maternal chronic medical conditions	Diabetes, hypertension, anaemia, asthma, thyroid disease	Improve control prior to conception and throughout pregnancy
	Nutritional	Undernutrition, micronutrient deficiencies	See following papers in supplement [66,67]
	Lifestyle/work related	Smoking, excess alcohol consumption, recreational drug use, excess physical work/activity	Behavior and community interventions targeting all women of childbearing age in general and for pregnant women in particular through antenatal care with early detection and treatment of pregnancy complications
	Maternal psychological health	Depression, violence against women	See following papers in supplement [66,67]
	Genetic and other	Genetic risk, e.g., family history Cervical incompetence Intra-uterine growth restriction Congenital abnormality	See following papers in supplement [66,67]
**Provider-initiated preterm birth:**	Medical induction or cesarean birth for:	Prior classical cesarean section, Placenta accrete.	In addition to the above: Programs and policies to reduce the practice of non-medically indicated induction of labor or cesarean birth
	obstetric indication Fetal indication Other - Not medically indicated	There is an overlap for indicated provider-initiated preterm birth with the risk factors for spontaneous preterm birth	

*Broad categories of possible interventions are listed here. They provide examples of possible interventions and not all the risk factors given in the examples are amenable to these interventions.

Spontaneous preterm birth is a multi-factorial process, resulting from the interplay of factors causing the uterus to change from quiescence to active contractions and to birth before 37 completed weeks of gestation. The precursors to spontaneous preterm birth vary by gestational age [24], and social and environmental factors, but the cause of spontaneous preterm labor remains unidentified in up to half of all cases [25]. Maternal history of preterm birth is a strong risk factor and most likely driven by the interaction of genetic, epigenetic and environmental risk factors [26]. Many maternal factors have been associated with an increased risk of spontaneous preterm birth, including young or advanced maternal age, short inter-pregnancy intervals and low maternal body mass index [27,28].

Another important risk factor is uterine over distension with multiple pregnancy. Multiple pregnancies (twins, triplets, etc.) carry nearly 10 times the risk of preterm birth compared to singleton births [29]. Naturally occurring multiple pregnancies vary among ethnic groups with reported rates from 1 in 40 in West Africa to 1 in 200 in Japan, but a large contributor to the incidence of multiple pregnancies has been rising maternal age and the increasing availability of assisted conception in high-income countries [30]. This has led to a large increase in the number of births of twins and triplets in many of these countries. For example, England and Wales, France and the United States reported 50 to 60% increases in the twin rate from the mid-1970s to 1998, with some countries (e.g. Republic of Korea) reporting even larger increases [31]. More recent policies, limiting the number of embryos transferred during in vitro fertilisation may have begun to reverse this trend in some countries [32], while others continue to report increasing multiple birth rates [33,34].

Infection plays an important role in preterm birth. Urinary tract infections, malaria, bacterial vaginosis, HIV and syphilis are all associated with increased risk of preterm birth [35]. In addition, other conditions have more recently been shown to be associated with infection, e.g., "cervical insufficiency" resulting from ascending intrauterine infection and inflammation with secondary premature cervical shortening [36].

Some lifestyle factors that contribute to spontaneous preterm birth include stress and excessive physical work or long times spent standing [28]. Smoking and excessive alcohol consumption as well as periodontal disease also have been associated with increased risk of preterm birth [35].

Preterm birth is both more common in boys, with around 55% of all preterm births occurring in males [37], and is associated with a higher risk of dying when compared to girls born at a similar gestation [38]. The role of ethnicity in preterm birth (other than through twinning rates) has been widely debated, but evidence supporting a variation in normal gestational length with ethnic group has been reported in many population-based studies since the 1970s [39]. While this variation has been linked to socioeconomic and lifestyle factors in some studies, recent studies suggest a role for genetics. For example, babies of black African ancestry tend to be born earlier than Caucasian babies [24,40]. However, for a given gestational age, babies of black African ancestry have less respiratory distress [41], lower neonatal mortality [42] and are less likely to require special care than Caucasian babies [24]. Babies with congenital abnormalities are more likely to be born preterm, but are frequently excluded from studies reporting preterm birth rates. Few national-level data on the prevalence of the risk factors for preterm birth are available for modelling preterm birth rates.

The number and causes of provider-initiated preterm birth are more variable. Globally, the highest burden countries have very low levels due to lower coverage of pregnancy monitoring and low caesarean birth rates (less than 5% in most African countries). However, in a recent study in the United States, more than half of all provider-initiated pre-term births at 34 to 36 weeks gestation were carried out in absence of a strong medical indication [43]. Unintended preterm birth also can occur with the elective delivery of a baby thought to be term due to errors in gestational age assessment [44]. Clinical conditions underlying medically-indicated preterm birth can be divided into maternal and fetal of which severe preeclampsia, placental abruption, uterine rupture, cholestasis, fetal distress and fetal growth restriction with abnormal tests are some of the more important direct causes recognized [39]. Underlying maternal conditions (e.g., renal disease, hypertension, obesity and diabetes) increase the risk of maternal complications (e.g., preeclampsia) and medically-indicated preterm birth. The worldwide epidemic of obesity and diabetes is, thus, likely to become an increasingly important contributor to global preterm birth. In one region in the United Kingdom, 17% of all babies born to diabetic mothers were preterm, more than double the rate in the general population [24]. Both maternal and fetal factors are more frequently seen in pregnancies occurring after assisted fertility treatments, thus increasing the risk of both spontaneous and provider-initiated preterm births [44,45].

Differentiating the causes of preterm birth is particularly important in countries where cesarean birth is common. Nearly 40% of preterm births in France and the United States were reported to be provider-initiated in 2000, compared to just over 20% in Scotland and the Netherlands. The levels of provider-initiated preterm births are increasing in all these countries in part due to more aggressive policies for caesarean section for poor foetal growth [46,47]. In the United States, this increase is reported to be at least in part responsible for the overall increase in the preterm birth rate from 1990 to 2007 and the decline in perinatal mortality [39]. No population-based studies are available from low- or middle-income countries. However, of the babies born preterm in tertiary facilities in low- and middle-income countries, the reported proportion of preterm births that were provider-initiated ranged from around 20% in Sudan and Thailand to nearly 40% in 51 facilities in Latin America and a teaching hospital in Ghana [48-51]. However, provider-initiated preterm births will represent a relatively smaller proportion of all preterm births in these countries where access to diagnostic tools is limited. These pregnancies, if not delivered electively, will follow their natural history, and may frequently end in spontaneous preterm birth (live or stillbirth)[52].

### Preterm birth--how is it measured?

There are many challenges to measuring preterm birth rates that have inhibited national data interpretation and multi-country assessment. In addition to the variable application of the definition, the varying methods used to measure gestational age and the differences in case ascertainment and registration complicate the interpretation of preterm birth rates across and within nations.

#### Assessing gestational age

Measurement of gestational age has changed over time. As the dominant effect of gestational age on survival and long-term impairment has become apparent over the last 30 years, perinatal epidemiology has shifted from measuring birthweight alone to focusing on gestational age. However, many studies, even of related pregnancy outcomes, continue to omit key measures of gestational age. The most accurate "gold standard" for assessment is routine early ultrasound assessment together with foetal measurements, ideally in the first trimester. Gestational assessment based on the date of last menstrual period (LMP) was previously the most widespread method used and remains the only available method in many settings. It assumes that conception occurs on the same day as ovulation (14 days after the onset of the LMP). It has low accuracy due to considerable variation in length of menstrual cycle among women, conception occurring up to several days after ovulation and the recall of the date of LMP being subject to errors [53]. Many countries now use "best obstetric estimate," combining ultrasound and LMP as an approach to estimate gestational age. The algorithm used can have a large impact on the number of preterm births reported. For example, a large study from a Canadian teaching hospital found a preterm rate of 9.1% when assessed using ultrasound alone, compared to 7.8% when using LMP and ultrasound [31].

Any method using ultrasound requires skilled technicians, equipment and for maximum accuracy, first-trimester antenatal clinic attendance. These are not common in low-income set tings where the majority of preterm births occur. Alternative approaches to LMP in these set tings include clinical assessment of the newborn after birth, fundal height or birthweight as a surrogate. While birthweight is closely linked with gestational age, it cannot be used interchangeably since there is a range of "normal" birthweight for a given gestational age and gender. Birthweight is likely to overestimate preterm birth rates in some settings, especially in South Asia where a high proportion of babies are small for gestational age.

#### Accounting for all births

The recording of births and deaths and the likelihood of active medical intervention after preterm birth are affected by perceptions of viability and social and economic factors, especially in those born close to the lower gestational age cut-off used for registration. Any baby showing signs of being live at birth should be registered as a livebirth regardless of the gestation [54]. The registration thresholds for stillbirths vary between countries from 16 to 28 weeks, and under-registration of both live and stillbirths close to the registration boundary is well documented [55]. The cut-off for viability has changed over time and varies across settings, with babies born at 22 to 24 weeks receiving full intensive care and surviving in some high-income countries, whilst babies born at up to 32 weeks gestation are perceived as non-viable in many low-resource settings. An example of this reporting bias is seen in high-income settings where the increase in numbers of extremely preterm (<28 weeks) births registered is likely to be due to improved case ascertainment rather than a genuine increase in preterm births in this group [56] and three community cohorts from South Asia with high overall preterm birth rates of 14 to 20%, but low proportions (2%) of extremely preterm births (<28 weeks) compared to the proportion from pooled datasets from developed countries (5.3%). In addition, even where care is offered to these very preterm babies, intensive care may be rationed [57,58].

Other cultural and social factors that have been reported to affect completeness of registration include provision of maternity benefits for any birth after the registration threshold, the need to pay burial costs for a registered birth but not for a miscarriage and increased hospital fees following a birth compared to a miscarriage [59]. In low-income settings, a live preterm birth may be counted as a stillbirth due to perceived non-viability or to "protect the mother" [55].

The definition of preterm birth focuses on live-born babies only. Counting all preterm births, both live and stillborn, would be preferable to improve comparability especially given stillbirth/livebirth misclassification. An increasing proportion of all preterm infants born will be stillborn with decreasing gestational age. The pathophysiology is similar for live and stillbirths; thus, for the true public health burden, it is essential to count both preterm babies born alive and all stillbirths [23]. Until these classification differences based on method (Table 3), lower gestational age cut-offs for registration of preterm birth, the use of singleton versus all births (including multiples), the inclusion of live births versus total births (including live and stillbirths) and case ascertainment have been resolved, caution needs to be applied when interpreting regional and temporal variations in preterm birth rates.

**Table 3:** Gestational age methods, accuracy and limitations

Method	Accuracy	Details	Availability/feasibility	Limitations
**Early ultrasound scan**	+/- 5 days if first trimester +/- 7 days after first trimester	Estimation of fetal crown-rump length +/- biparietal diameter/femur length between gestational age 6 - 18 weeks	Ultrasound not always available in low-income settings and rarely done in first trimester	May be less accurate if fetal malformation, or maternal obesity
**Fundal Height**	~ +/- 3 weeks	Distance from symphysis pubis to fundus measured with a tape measure	Feasible and low cost	In some studies similar accuracy to LMP Potential use with other variables to estimate GA when no other information available
**Last menstrual period**	~ +/- 14 days	Women's recall of the date of the first day of her last menstrual period	Most widely used	Lower accuracy in settings with low literacy. Affected by variation in ovulation and also by breastfeeding. Digit preference
**Birthweight as a surrogate of gestational age**	More sensitive/specific at lower gestational age e.g. <1500 g most babies are preterm		Birthweight measured for around half of the world's births	Requires scales and skill. Digit preference
**Newborn examination**	~ +/- 13 days for Dubowitz, higher range for all others	Validated scores using external +/or neurological examination of the newborn e.g. Parkin, Finnstrom, Ballard and Dubowitz scores	Mainly specialist use so far. More accurate with neurological criteria which require considerable skill. Potential wider use for simpler scoring systems	Accuracy dependant on complexity of score and skill of examiner. Training and ongoing quality control required to maintain accuracy
**Best obstetric estimate**	Around +/- 10 days (between ultrasound and newborn examination)	Uses an algorithm to estimate gestational age based on best information available	Commonly used in high-income settings	Various algorithms in use, not standardized

Adapted from Parker, Lawn and Stanton (unpublished Master's thesis)

## Using the data for action

### Preterm birth rates--where, and when?

#### Global, regional and national variation of preterm birth for the year 2010

**Figure 3:**
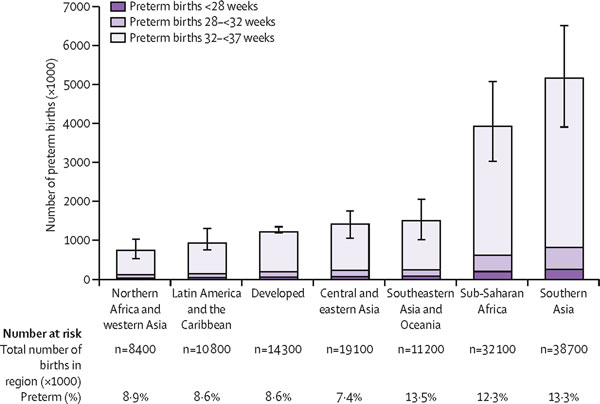
**Preterm births by gestational age and region for the year 2010**. Based on Millennium Development Goal regions. Source: Reproduced with permission from Blencowe et al. (2012) National, regional and worldwide estimates of preterm birth rates in the year 2010 with time trends since 1990 for selected countries: a systematic analysis and implications. Lancet **379**(9832): 2162-2172.

New WHO estimates of global rates of preterm births indicate that of the 135 million live births worldwide in 2010, 14.9 million babies were born preterm, representing a preterm birth rate of 11.1% [14]. Over 60% of preterm births occurred in sub-Saharan Africa and South Asia where 9.1 million births (12.8%) annually are estimated to be preterm (Figure 3). The high absolute number of preterm births in Africa and Asia are related, in part, to high fertility and the large number of births in those two regions in comparison to other parts of the world.

The variation in the rate of preterm birth among regions and countries is substantial and yield a different picture to other conditions in that some high-income countries have very high rates. Rates are highest on average for low-income countries (11.8%), followed by lower middle-income countries (11.3%) and lowest for upper middle- and high-income countries (9.4% and 9.3%). However, relatively high preterm birth rates are seen in many individual high-income countries where they contribute substantially to neonatal mortality and morbidity. Of the 1.2 million preterm births estimated to occur in high-income regions, more than 0.5 million (42%) occur in the United States. The highest rates by Millennium Development Goal Regions [60] are found in Southeastern and South Asia where 13.4% of all live births are estimated to be preterm (Figure 3).

The uncertainty ranges in Figure 3 are indicative of another problem -- the huge data gaps for many regions of the world. Although these data gaps are particularly great for Africa and Asia, there also are gaps in data from high-income countries. While data on preterm birth-associated mortality are lacking in these settings, worldwide there are almost no data currently on acute morbidities or long-term impairment associated with prematurity, thus preventing even the most basic assessments of service needs.

**Figure 4:**
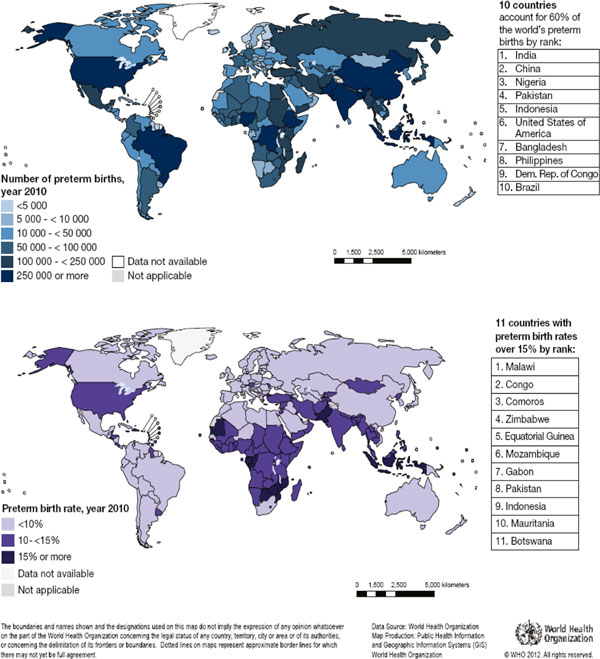
**Preterm births in 2010**. Source: Blencowe, H., et al. (2012) **Chapter 2: 15 million preterm births: Priorities for action based on national, regional and global estimates**. In *Born Too Soon: the Global Action Report on Preterm Birth*. http://www.who.int/pmnch/media/news/2012/borntoosoon_chapter2.pdf 2012 [79]. Not applicable = non WHO Members State.

The maps in Figure 4 depict preterm birth rates and the absolute numbers of preterm birth in 2010 by country. Estimated rates vary from around 5 in several Northern European countries to 18.1% in Malawi. The estimated preterm birth rate is less than 10% in 88 countries, whilst 11 countries have estimated rates of 15% or more (Figure 4). The 10 countries with the highest numbers of estimated preterm births are India, China, Nigeria, Pakistan, Indonesia, United States, Bangladesh, the Philippines, Democratic Republic of the Congo and Brazil (Figure 4). These 10 countries account for 60% of all preterm births worldwide.

Mortality rates increase with decreasing gestational age, and babies who are both preterm and small for gestational age are at even higher risk [61]. Babies born at less than 32 weeks represent about 16% of all preterm births [14]. Across all regions, mortality and morbidity are highest among those babies although improvements in medical care have led to improved survival and long-term outcomes among very and extremely preterm babies in high-income countries [62]. In 1990, around 60% of babies born at less than 28 weeks gestation survived in high-income settings, with approximately two-thirds surviving without impairment [63]. In these high-income countries, almost 95% of those born at 28 to 32 weeks survive, with more than 90% surviving without impairment. In contrast, in many low-income countries, only 30% of those born at 28 to 32 weeks survive, with almost all those born at <28 weeks dying in the first few days of life. In all settings, these very or extremely preterm babies account for the majority of deaths, especially in low-income countries where even simple care is lacking [64].

#### Preterm births time trends 1990 to 2010

Absolute numbers and rates of preterm birth for 65 countries in Europe, the Americas and Australasia from 1990 to 2010 for these countries suggest an increasing burden of preterm birth [5]. This increase is partly explained by an increase in preterm births occurring at 32 to <37 weeks (late and moderate preterm) reported over the past decades in some countries [65]. Despite a reduction in the number of live births, the estimated number of preterm births in these countries increased from 2.0 million in 1990 to nearly 2.2 million in 2010 [14]. Preterm birth rate trends for low- and middle-income countries suggest an increase in some countries (e.g., China) and some regions (e.g., South Asia) but given changes in the data type and the measurement of gestational age, these remain uncertain.

#### Priority policy and program actions based on the data

In 2010, approximately 15 million babies were born preterm, and more than 1 million died due to complications in the first month of life, more from indirect effects, and millions have a lifetime of impairment. The burden of preterm birth is highest in low-income countries, particularly those in South Asia. Yet unlike many other global health issues, preterm birth is truly a global problem with a high burden being found in high-income countries as well (e.g. the United States where almost 1 in 8 babies is preterm). However, while the risk of preterm birth is high for both the poorest and the richest countries, there exists a major survival gap in some regions for babies who are preterm. In high-income settings, half of babies born at 24 weeks may survive, but in low-income settings half of babies born at 32 weeks still die due to a lack of basic care [64].

Preterm birth rates appear to be increasing in most of the countries where data are available. Some of this increase may be accounted for by improved registration of the most preterm babies associated with increased viability and by improved gestational assessment, with change to near universal ultrasound for dating pregnancies in these settings. It may, however, represent a true increase. Possible reasons for this include increases in maternal age, access to infertility treatment, multiple pregnancies and underlying health problems in the mother, especially with increasing age of pregnancy and changes in obstetric practices with an increase in provider-initiated preterm births in moderate and late preterm infants who would not have otherwise been born preterm [46]. In the 1980s and 1990s, the increases seen in many high-income countries were attributed to higher multiple gestation and preterm birth rates amongst assisted conceptions after treatment for sub-fertility. Recent changes in policies limiting the number of embryos that can be implanted have led to a reduction in preterm births due to assisted fertility treatments in many countries [63]. However, in many middle-income regions with newer, relatively unregulated assisted fertility services, a similar increase may be seen if policies to counteract this are not introduced and adhered to. A reduction in preterm birth was reported from the 1960s to 1980s in a few countries (e.g. Finland, France, Scotland), and this was attributed, in part, to improved socioeconomic factors and antenatal care. For the majority of countries in low- and middle-income regions, it is not possible to estimate trends in preterm birth over time as there are not sufficient data to provide reliable evidence of a time trend for preterm birth overall. Some countries in some regions (e.g. South and Eastern Asia) have data suggesting possible increases in preterm birth rates over time, but this may represent measurement artifact due to increases in data and data reliability.

Distinguishing spontaneous and provider-initiated preterm birth is of importance to programs aiming to reduce preterm birth. For spontaneous preterm births, the underlying causes need to be understood and addressed while in the case of provider-initiated preterm births both the underlying conditions (e.g. preeclampsia) and obstetric policies and practices require assessment and to be addressed [66,67].

The proportion of neonatal deaths attributed to preterm births is inversely related to neonatal mortality rates, because in countries with very high neonatal mortality, more deaths occur due to infections such as sepsis, pneumonia, diarrhea and tetanus as well as to intrapartum-related "birth asphyxia" [2]. However, although the proportion of deaths due to preterm birth is lower in low-income countries than in high-income countries, the cause-specific rates are much higher in low- and middle-income than in high-income countries. For example, in Afghanistan and Somalia, the estimated cause-specific rate for neonatal deaths directly due to preterm birth is 16 per 1,000 compared to Japan, Norway and Sweden where it is under 0.5 per 1,000. This is due to the lack of even simple care for premature babies resulting in a major survival gap for babies depending on where they are born [64].

Preterm birth can result in a range of long-term complications in survivors, with the frequency and severity of adverse outcomes rising with decreasing gestational age and decreasing quality of care (Table 1). Most babies born at less than 28 weeks need neonatal intensive care services to survive, and most babies 28 to 32 weeks will need special newborn care at a minimum. The availability and quality of these services are not yet well established in many low- and middle-income countries. Many middle-income countries, currently scaling up neonatal intensive care, are just beginning to experience these long-term consequences in survivors. 43% of the estimated 0.9 million preterm babies surviving with neurodevelopmental impairment are from middle income countries [8]. These effects are most marked amongst survivors born extremely preterm; however, there is increasing evidence that all premature babies regardless of gestational age are at increased risk. The vast majority (84%) of all preterm births occur at 32 to 36 weeks. Most of these infants will survive with adequate supportive care and without needing neonatal intensive care. However, even babies born at 34 to 36 weeks have been shown to have an increased risk of neonatal and infant death when compared with those born at term and contribute importantly to overall infant deaths [68]. Babies born at 34 to 36 weeks also experience increased rates of short-term morbidity associated with prematurity (e.g., respiratory distress and intraventricular hemorrhage) than their peers born at term [69-71]. In the longer term, they have worse neurodevelopmental and school performance outcomes and increased risk of cerebral palsy [72,73]. On a global level, given their relatively larger numbers, babies born at 34 to 36 weeks are likely to have the greatest public health impact and to be of the most importance in the planning of services (e.g., training community health workers in Kangaroo Mother Care (KMC), essential newborn care and special care of the moderately preterm baby) [64].

We have highlighted the differences in preterm birth rates among countries, but marked disparities are also present within countries. For example, in the United States in 2009, reported preterm birth rates were as high as 17.5% in black Americans, compared to just 10.9% in white Americans, with rates varying from around 11 to 12% in those 20 to 35 years of age to more than 15% in those under age 17 or over 40 [13]. Disparities within countries need to be better understood in order to identify high-risk groups and improve care.

The economic costs of preterm birth are large in terms of the immediate neonatal intensive care and ongoing long-term complex health needs frequently experienced. These costs, in addition, are likely to rise as premature babies increasingly survive at earlier gestational ages in all regions. This survival also will result in the increased need for special education services and associated costs that will place an additional burden on affected families and the communities in which they live [74]. An increased awareness of the long-term consequences of preterm birth (at all gestational ages) is required to fashion policies to support these survivors and their families as part of a more generalised improvement in quality of care for those with disabilities in any given country. In many middle-income countries, preterm birth is an important cause of disability. For example, a third of all children under 10 in schools for the visually impaired in Vietnam and more than 40% of under-5's in similar schools in Mexico have blindness secondary to retinopathy of prematurity [75,76].

#### Actions to improve the data

The estimates from the Born Too Soon report represent a major step forward in terms of presenting the first-ever national preterm birth estimates [77]. However, action is required to improve the availability and quality of data from many countries and regions and, where data are being collected and analysed, to improve consistency among countries. These are vital next steps to monitor the progress of policies and programs aimed at reducing the large toll of preterm birth (Table 4). Efforts in every country should be directed to increasing the coverage and systematic recording of all preterm births in a standard reporting format. Standardisation of the definition in terms of both the numerator (the number of preterm births) and the denominator (the number of all births) is essential if trends and rankings are to be truly comparable. Collecting data on both live and stillbirths separately will allow further quantification of the true burden, while data focusing on live births only are required for monitoring of neonatal and longer-term outcomes. These estimates indicate the large burden amongst live-born babies. However, in developed countries with available data, between 5 and 10% of all preterm births are stillbirths, and the figure may be higher in countries with lower levels of medicallyinduced preterm birth. Distinguishing between live births and stillbirths may vary depending on local policies, the availability of intensive care and perceived viability of babies who are extremely preterm. If estimates for live-born preterm babies were linked to estimates for stillbirths, this would improve tracking among countries and over time. Achieving consensus around the different types of preterm birth and comparable case definitions, whilst challenging, are required where resources allow to further understand the complex syndrome of preterm birth [23].

**Table 4:** Actions to improve national preterm birth rate data

**Definition consistency**	**Numerator (number of preterm births)**
Consensus on definition of preterm birth for international comparison, specifying gestational age	Simplified, lower cost, consistent measures of gestational age (GA) Widespread use and recording of GA
	Consistent inclusion of all live births of all gestations or weight, and noting if singleton or multiple births and noting the proportion that are under 500 g/22 weeks and under 1,000 g/28 weeks for international comparison Also record all stillbirths from 500 g/22 weeks and 1,000 g/28 weeks (whilst collecting by other national definition for stillbirth if different e.g., 20 weeks in United States)
	**Denominator (number of births)**
	Consistent measurement of all live births of all gestations noting if less than 22 weeks and if singleton or multiple births
	Also record all stillbirths
	
**Actions to improve the data**	**Focus on capture and consistency:**
	Gestational age and birthweight recording for all births
	Improve reporting of neonatal cause of death with preterm as direct cause and as risk factor (counting deaths of preterm babies who die from other causes)
	Collection of impairment data e.g., cerebral palsy and retinopathy of prematurity (ROP) rates according to a basic minimum dataset to increase consistency
	For settings where additional capacity available:
	Improve measurement e.g. gestational age assessment using early, high-quality ultrasound scan, development and refinement of improved gestational age assessment tools for use in low-resource settings
	**Increase the granularity of the data:**
	Record if provider-initiated, e.g., cesarean birth, or spontaneous and the basic phenotype, e.g. infection/relative contribution of each cause especially multiple births
	Improve the linkage of data to action: e.g., collating data by gender, socioeconomic status, ethnicity, subnational e.g. state
	Impairment data according to a more comprehensive standard dataset
**Data for action**	Set goals for national and global level for
	1. Reduction of deaths amongst preterm babies by 2025
	2. Reduction of preterm birth rates by 2025
	Regular reporting of preterm birth rates and preterm-specific mortality rates at national level and to global level to track against goals

Note that weight is the preferred measure in ICD 10, but GA is commonly used now. The weight and GA "equivalents" are approximate.

In many low- and middle-income countries without wide-scale vital registration, no nationally representative data are available on rates of preterm birth. Substantial investment and attention are required to improve vital registration systems and to account for all birth outcomes [78]. In the meantime, the amount of population-based data available in high-burden countries could be dramatically increased to better inform future estimates and monitor time trends if data on preterm birth rates were able to be included in nationally representative surveys such as the Demographic and Health Surveys (DHS), but this will require developing, testing and training in the use of preterm-specific survey-based tools which are not currently available. The advent of inexpensive portable ultrasound machines makes inclusion of routine early ultrasound scans in demographic surveillance sites or representative cohorts a promising route to increase data availability in these settings in the short term. Innovation for simpler, low-cost, sensitive and specific tools for assessing gestational age could improve both the coverage and quality of gestational age assessment. Data from hospital-based information systems would also be helpful, but potential selection and other biases must be taken into account. Simpler standardized tools to assess acute and long-term morbidities-associated preterm birth also are critically important to inform program quality improvement to reduce the proportion of survivors with preventable impairment.

## Conclusion

There are sufficient data to justify action now to reduce this large burden of 15 million preterm births and more than one million neonatal deaths. Innovative solutions to prevent preterm birth and hence reduce preterm birth rates all around the world are urgently needed. This also requires strengthened data systems to adequately track trends in preterm birth rates and program effectiveness. These efforts must be coupled with action now to implement improved antenatal, obstetric and newborn care to increase survival and reduce disability amongst those born too soon. These are reviewed further in the following papers in this supplement.

## List of abbreviations used

pPROM: prelabour premature rupture of membranes; WHO: World Health Organization.

## Competing interests

The author's declare that they have no conflict of interest. The authors alone are responsible for the views expressed in this article and they do not necessarily represent the views, decisions or policies of the institutions with which they are affiliated.

## Authors' contribution

HB, MK and JL drafted the paper with SC, DC, MZO, LS, ABM. All authors reviewed the final manuscript.

## Funding

HB and SC were funded through a grant from the Bill & Melinda Gates Foundation through the Child Health Epidemiology Reference Group. JL and MK were funded by the Bill & Melinda Gates Foundation though Save the Children's Saving Newborn Lives program.

## Supplementary Material

Additional file 1 **In line with the journal's open peer review policy, copies of the reviewer reports are included as additional file **1. Click here for file
