# Supplementary material for: Born Too Soon: The global epidemiology of 15 million preterm births
Source: Reprod Health. 2013 Nov 15;10(Suppl 1):S2. doi: 10.1186/1742-4755-10-S1-S2 (PMC3828585; doi:10.1186/1742-4755-10-S1-S2)
Supplement: Additional file 1 — In line with the journal's open peer review policy, copies of the reviewer reports are included as additional file 1. [file 1742-4755-10-S1-S2-S1.doc]

**Referee’s comments to the authors– this sheet WILL be seen by the author(s) and published with the article**

| Title | **15 million preterm births: priorities for action based on national, regional and global estimates** |
| --- | --- |
| Author(s) | Hannah Blencowe, Simon cousens, Doris Chou, Mikkel Oestergaard, Lale Say, Ann-Beth Moller, Mary Kinney and Joy Lawn |
| Referee’s name | Cathy Spong |

**When assessing the work, please consider the following points, where applicable:**

1. Is the question posed by the authors new and well defined?
2. Are the methods appropriate and well described, and are sufficient details provided to replicate the work?
3. Are the data sound and well controlled?
4. Does the manuscript adhere to the relevant standards for reporting and data deposition?
5. Are the discussion and conclusions well balanced and adequately supported by the data?
6. Do the title and abstract accurately convey what has been found?
7. Is the writing acceptable?

Please make your report as constructive and detailed as possible in your comments so that authors have the opportunity to overcome any serious deficiencies that you find and please also divide your comments into the following categories:

- Major Compulsory Revisions (which the author must respond to before a decision on publication can be reached)
- Minor Essential Revisions (such as missing labels on figures, or the wrong use of a term, which the author can be trusted to correct)
- Discretionary Revisions (which are recommendations for improvement but which the author can choose to ignore)

Where possible please supply references to substantiate your comments.

When referring to the manuscript please provide specific page and paragraph citations where appropriate.

This manuscript is very comprehensive and covers a lot of information as well as explains many of the nuances of the data that are important for the reader to understand (eg the limitations of available data, difficulty of collecting reliable information on stillbirths, implications of having different cutoffs for preterm birth across countries).

Overall much of the material in this manuscript is covered in other manuscripts that have been published – the material is not new or novel – which does not detract from its importance, however it is not actually clear to me as the reader what the purpose of this manuscript is. There is no objective stated, either in the abstract or in the manuscript itself, which left me wondering what was the main purpose of writing the paper. I believe I understand this is part of a supplement or a group of papers, so it may be more clear in that context. Having now read the manuscript several times, I think the point is in Table 4 and on page 11 (actions to improve the data), if so I think this could be strengthened some and the prior 10 pages could likely be condensed, as much of this material is review.

The title is not as reflective of the paper as one might want – perhaps it could be revised.

Table 2:

-obesity is controversial on whether it is a risk factor for PTB

-prevention and treatment of infections such as chorioamnionitis and BV prior to pregnancy have not been shown to reduce PTB. Treatment of chorionamnionitis or asymptomatic BV also have not been shown to prevent preterm birth or improve outcome.

-provider indicated preterm birth examples could be placenta accreta, prior classical cesarean

-Would want to also reduce the practice of non-medically indicated vaginal birth (inductions)

Table 3:

Severe growth restriction doesn’t typically manifest in the first trimester which is why ultrasound then is best to determine gestational age

*(continue on the next sheet)*

**Referee’s comments to the authors– this sheet WILL be seen by the author(s) and published with the article**

| Title | **15 million preterm births: priorities for action based on national, regional and global estimates** |
| --- | --- |
| Author(s) | Hannah Blencowe, Simon Cousens, Doris Chou, Mikkel Oestergaard, Lale Say, Ann-Beth Moller, Mary Kinney and Joy Lawn |
| Referee’s name | Joanne Katz |

**When assessing the work, please consider the following points, where applicable:**

1. Is the question posed by the authors new and well defined?
2. Are the methods appropriate and well described, and are sufficient details provided to replicate the work?
3. Are the data sound and well controlled?
4. Does the manuscript adhere to the relevant standards for reporting and data deposition?
5. Are the discussion and conclusions well balanced and adequately supported by the data?
6. Do the title and abstract accurately convey what has been found?
7. Is the writing acceptable?

Please make your report as constructive and detailed as possible in your comments so that authors have the opportunity to overcome any serious deficiencies that you find and please also divide your comments into the following categories:

- Major Compulsory Revisions (which the author must respond to before a decision on publication can be reached)
- Minor Essential Revisions (such as missing labels on figures, or the wrong use of a term, which the author can be trusted to correct)
- Discretionary Revisions (which are recommendations for improvement but which the author can choose to ignore)

Where possible please supply references to substantiate your comments.

When referring to the manuscript please provide specific page and paragraph citations where appropriate.

**General comments:** This is a paper that reviews the epidemiology of preterm birth, including risk factors, mortality and health consequences for infants who survive. The review also tackles rends in prevalence and intervention priorities. The perspective is global but the emphasis is on low and middle income countries as their data capture and health systems are in need of resources, while the burden in absolute and relative terms is high.

The paper is well written and makes for easy reading. The figures and tables are helpful and informative.

**Major compulsory revisions:**

**Minor essential revisions:** My only minor comment is about improving the data obtained from the Demographic and Health Surveys for use in tracking trends. Given the cross-sectional nature of those surveys and the lengthy recall about reproductive outcomes, I cannot see this mode of data collection as being a mechanism by which to improve accuracy of data collection on preterm births. Sentinal or representative cohorts where early ultrasound measure could be obtained might make more sense, although this type of data collection has costs, even with the advent of cheaper, portable ultrasound devices.

## Comments from the Editor:

For all the articles, these should be seen as independent papers that can be read without the need to read the other papers in the series.
